# Synthesis, Structural Characterization, and Magnetic Properties of Two New Fe(III)Mn(III) 1D Bimetallic Compounds

**DOI:** 10.1021/acsomega.4c09887

**Published:** 2025-02-24

**Authors:** Abdelwahab Hassan, Eslam Hemida, Nada I. Mahmoud, Mohamed Kamel, Hany M. Elsharkawy, Ahmed S. G. Khalil, Mahmoud Abdel-Hafiez, Mohamed R. Saber

**Affiliations:** † Physics Department, Faculty of Science, Fayoum University, Fayoum 63514, Egypt; ‡ Science Department, Rustaq College of Education, University of Technology and Applied Sciences, PO Box 10, Al Rustaq 329, Sultanate of Oman; § Chemistry Department, Faculty of Science, Fayoum University, Fayoum 63514, Egypt; ∥ Environmental and Smart Technology Group, Faculty of Science, Fayoum University, Fayoum 63514, Egypt; ⊥ Department of Applied Physics & Astronomy, University of Sharjah, PO Box 27272 Sharjah, UAE; # Department of Physics and Astronomy, Uppsala University, Uppsala 751 20, Sweden

## Abstract

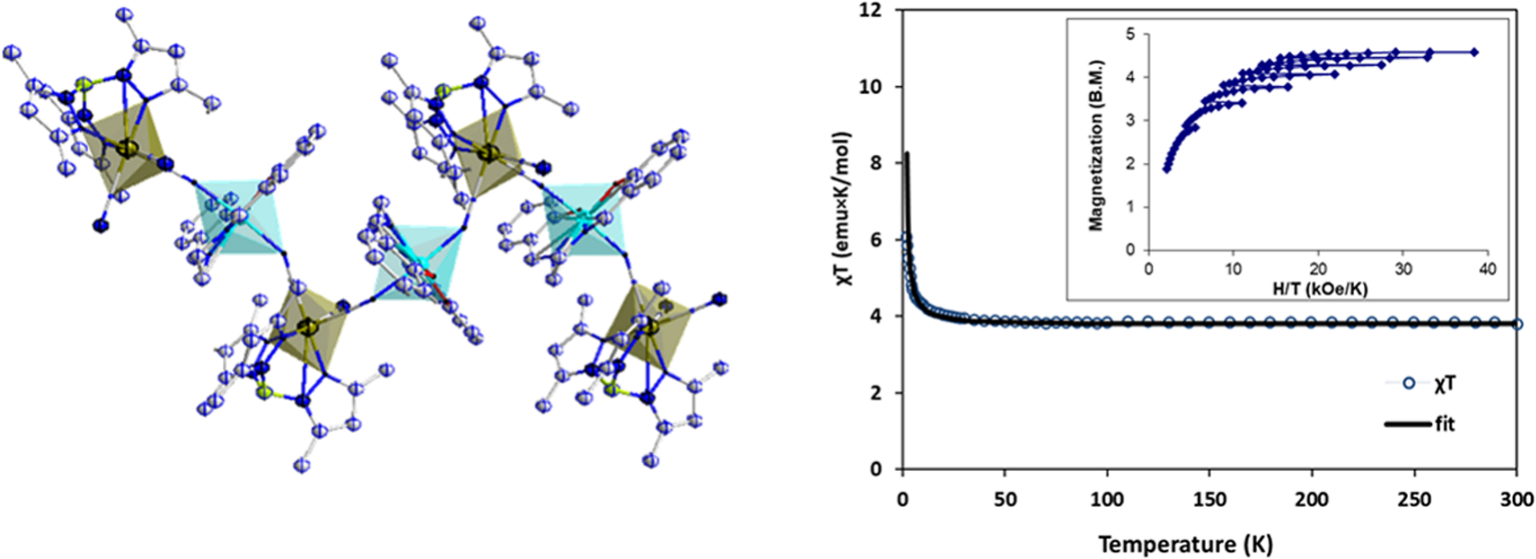

Two Fe(III)Mn(III) bimetallic compounds, [Tp*Fe (CN)_3_][(Mn^III^salen)]·MeOH·MeCN (**1**) and [Tp*Fe (CN)_3_][Mn^III^salphen]·MeOH·H_2_O (**2**), were prepared by assembling the tricyanometalate building block (TEA)_4_N [Tp*Fe(CN)_3_] [Tp* = hydrotris(3,5-dimethylpyrazol-1-yl)borate] and the respective Mn Schiff base precursors [salen = *N*,*N*′-ethylenebis(salicylideneiminate), salphen = *N*,*N*-bis(salicylidene)-1,2-phenylenediamine]. Both compounds exhibit a one-dimensional (1D) zigzag chain structure linked by cyanide bridges, forming a (−Fe–C≡N–Mn–N≡C−)*_n_* motif. Magnetic studies show a gradual increase of χ_M_*T* values in both complexes upon lowering temperature, indicating ferromagnetic coupling between the Fe^III^ and Mn^III^ metal centers with Curie–Weiss constants of +1.1 K in (**1**) and +1.0 K in (**2**). Ferromagnetic interactions are attributed to the significantly bent Mn–NC angles (148.52 and 152.99° for (**1**) and 154.9 and 151.3° for (**2**)). The formation of 1D chains in the presence of MeOH challenges earlier reports that linked chain formation to the absence of MeOH in the reaction medium. This finding highlights the highly sensitive nature of this reaction system to various factors, including the influence of solvents on intermolecular interactions, the coordinative properties and polarity of the solvent, the steric and electronic characteristics of the precursors, and specific reaction conditions, such as temperature, concentration, and molar ratios.

## 1 Introduction

The predominant focus in molecular magnetic materials recent research revolves around the systematic control of structural features to modulate the type and magnitude of magnetic anisotropy and magnetic coupling to engender interesting magnetic behaviors such as single-chain magnets (SCMs), single-molecule magnets (SMMs), high-Tc magnets, and multifunctional magneto-optical/conductive materials.^1−8^ In this vein, cyano-bridged bimetallic assemblies have been the subject of great interest because of their interesting structural and magnetic features that make them suitable for designing new materials.^9−17^ The cyanide ligand can effectively mediate magnetic interactions between paramagnetic centers predictably because of its linear coordination mode and expected orthogonality with magnetic orbitals on the bridged magnetic centers.^18,19^ The molecular precursors [Tp^R^Fe (CN)_3_]^−^ have been used to design and synthesize a variety of magnetic materials with different dimensionalities and structures leading to interesting magnetic properties.^15,20−24^ Despite the predesigned structure and directional coordination modes of these building blocks, however, their reactions have been proven to be quite sensitive to different factors, including reaction medium, temperature, and second reactant structural features. The reaction of [TpFe(CN)_3_]^−^ with [Mnsalen(H_2_O)]_2_(ClO_4_)_2_ in a volume ratio of 1:1 MeCN/H_2_O at room temperature has led to the formation of the tetranuclear cluster [(Tp*)Fe(CN)_3_]_2_[Mn(salen)]_2_·H_2_O with linear Mn–NC angles.^18^ Using [Mn(TTF-salphen)][OAc] TTF-salphen^2–^ = 2,2′-((2-(4,5-bis(methylthio)-1,3-dithiol-2-ylidene)-1,3-benzodithiole-5,6-diyl)bis(nitrilomethylidyne) bis(phenolate) dianion) in the MeOH/MeCN mixture produced dinuclear complex [(Tp)Fe(CN)_3_Mn(TTF-salphen)·CH_3_OH] with antiferromagnetic coupling.^25^ The reaction with [Mn(diphenylsalen)(H_2_O)_2_](ClO_4_) in MeOH (for Tp) or MeCN (for Tp*) produced one-dimensional (1D) chains: [Mn (diphenylsalen)Fe(Tp)(CN)_3_]*_n_* and [Mn (diphenylsalen)Fe(Tp*)(CN)_3_]·[2H_2_O]*_n_* with significantly bent Mn–NC angles leading to ferromagnetic coupling between the Fe^III^ and Mn^III^ ions.^26^ An alternating zigzag chain {[(Tp*)Fe(CN)_3_Mn(salcyen)]·H_2_O·2CH_3_OH}*_n_* [salcyen = *N*,*N*′- (1,2-cyclo hexanediylethylene) bis (salicylideneiminato) dianion] was also reported, showing spin-canting with dominant antiferromagnetic interactions.^18^ The sensitivity of this reaction system to solvents was demonstrated in the reaction with [(2-acnapen)(H_2_O)]Cl Schiff base [2-acnapen = *N*,*N*′ethylenebis(1-hydroxy-2-aceto naphthylidene iminato) dianion]. Conducting the reaction in H_2_O/MeCN/MeOH leads to dimeric [(Tp)Fe(CN)_3_][Mn(2-acnapen)(MeOH)]·MeOH, whereas using H_2_O/MeCN (1:1 v/v) leads to one-dimensional (1D) zigzag chains. The authors attributed the different reaction pathways to solvent effects on intermolecular interactions such as face-to-face π–π contacts, edge-to-face CH-π forces, and hydrogen bonds that led to stabilizing the dimeric structure in the presence of MeOH, which blocks the evolution toward higher dimensionality 1D structures due to its coordinative nature. In the absence of MeOH, the reaction proceeds toward higher dimensionality products with less intermolecular interactions.^27^ It is evident from the aforementioned examples that such rich structural versatility in this reaction system leads to significant variations in the type and magnitude of magnetic exchange interactions and, hence, the observed magnetic behavior of the resultant complexes. Herein, we report the syntheses, crystal structures, and magnetic properties of two 1D cyano-bridged heterobimetallic chain complexes, [Tp*Fe (CN)_3_Mn^III^salen]·MeOH·MeCN (**1**) and [Tp*Fe (CN)_3_Mn^III^salphen]·MeOH·H_2_O (**2**), produced from the MeOH:MeCN reaction medium. The magnetic studies demonstrated that the two compounds exhibit ferromagnetic coupling between the Fe^III^ and Mn^III^ ions.

## 2 Experimental Section

### 2.1 Physical Measurements

Infrared spectra were recorded by using a Bruker Invenio R FTIR spectrometer equipped with a Platinum ATR accessory. A Bruker D8 QUEST instrument was used to collect single-crystal X-ray diffraction (SC-XRD). The structure was solved and refined using the Bruker SHELXTL Software Package. The figures and pictures of the structure were drawn by using Diamond 4.6.8 Software. Elemental analysis was made using a VARIO EL III CHNS Element Analyzer. Magnetic measurements were conducted on crushed polycrystalline samples by using a Quantum Design PPMS instrument. The temperature dependence of the magnetization in the range of 2–300 K was recorded.

### 2.2 Materials

Salphen and salen Schiff bases were synthesized according to the literature^28−31^ from the condensation of salicylaldehyde with *O*-phenylenediamine or ethylene diamine, respectively. Mn^III^ Schiff base and tricyanoferrate precursors were synthesized according to the literature.^20,32,33^ All of the following reagents were commercially available and used without further purification: ethylene diamine (LOBA, 99%); KTp* (TCI America); ferric chloride hexahydrate (LOBA, 97%); ethanol (Sigma, 99%); methanol (Carlo Erba, 99%); tetraethylammonium chloride (Merck, 98%); acetonitrile, KCN, and other chemicals and solvents were of analytical grade.

#### 2.2.1 Synthesis of [Tp*Fe (CN)_3_Mn^III^salen]·MeOH·MeCN (**1**)

(Et)_4_N[Tp*Fe(CN)_3_] (0.2 mmol, 116 mg) in 5 mL of MeCN was added to [Mn^III^salen(H_2_O)]ClO_4_ (0.14 mmol, 62 mg) in 5 mL of MeOH to get a reddish-brown solution. The solution was left undisturbed for 2 days in order to obtain reddish-brown crystals. The crystals were filtered off, washed with 1 mL of cold MeOH (3 times), and air-dried (yield = 23%, 26 mg). Elemental analysis for C_37_H_42_BFeMnN_12_O_3_: anal. calcd, MW: 824.42. C, 53.91; H, 5.14; B, 1.31; Fe, 6.77; Mn, 6.66; N, 20.39; O, 5.82%. Found C, 53.82; H, 5.08; N, 20.46%. IR (KBr, cm^–1^): 457, 594, 628, 644, 752, 767, 796, 856, 904, 975, 1062, 1087, 1128, 1151, 1201, 1290, 1334, 1367, 1414, 1446, 1540, 1600, 1621 (ν_as_ C=N), 2138 (ν_as_ C≡N), 2358, 2537 (ν_as_ BH).

#### 2.2.2 Synthesis of [Tp*Fe (CN)_3_Mn^III^salphen]·MeOH·H_2_O (**2**)

Compound **(2)** was synthesized by adding [Mn^III^salphen(H_2_O)_2_]ClO_4_ (0.14 mmol, 70 mg) in 5 mL of MeOH to 0.2 mmol (116 mg) (Et)_4_N[Tp*Fe(CN)_3_] in 5 mL of acetonitrile (MeCN). The solution was left undisturbed for 2 days to get reddish-brown crystals (yield = 21%, 24 mg). Elemental analysis for C_39_ H_41_BFeMnN_11_O_4_: anal. calcd, MW: 849.42. C, 55.15; H, 4.87; B, 1.27; Fe, 6.57; Mn, 6.47; N, 18.14; O, 7.53%. Found C, 55.24; H, 4.79; N, 18.21%. IR (KBr, cm^–1^): 541, 630, 750, 811, 870, 1128, 1149. 1197, 1315, 1376, 1538, 1596, 1604, 2073 (ν_as_ C≡N), and 2140 (ν_as_ C≡N).

## 3 Results and Discussion

### 3.1 Syntheses and Structural Studies

Manganese Schiff base and tricyanoferrate precursors were synthesized following literature procedures.^32−34^ The reaction of [(Tp*)Fe(CN)_3_]^−^ with the corresponding Mn(III) Schiff bases in MeOH:MeCN (1:1 v/v) medium produced 1D zigzag chains [Tp*Fe (CN)_3_Mn^III^salen]·MeOH·MeCN (**1**) and [Tp*Fe(CN)_3_Mn^III^salphen]·MeOH·H_2_O (**2**) as elucidated using different spectroscopic and structural characterization techniques. IR spectra show strong peaks at 2138 cm^–1^ (**1**) and 2140 cm^–1^ (**2**), which can be attributed to the asymmetric stretching mode for C≡N.^35^ This is a different outcome compared to previously reported tricyanoferrate/Mn(III)SB reaction systems.^18,20,25−27^ For example, the same reaction between [Tp*Fe (CN)_3_]^−^ and [Mnsalen(H_2_O)]_2_(ClO_4_)_2_ in 1:1 MeCN/H_2_O produced a tetranuclear cluster [(Tp*)Fe(CN)_3_Mn(salen)]_2_·H_2_O.^18^ Similarly, several salen-based precursors led to the formation of tetranuclear assemblies with [TpFe (CN)_3_]^−^ such as [(Tp)Fe(CN)_3_Mn(acphen)]_2_,^20^ [(Tp)Fe(CN)_3_Mn(5-Bracphen)]_2_,^20^ and [(Tp)Fe(CN)_3_Mn(salen)]_2_·6H_2_O^20^ in different solvent combinations (MeOH/MeCN/H_2_O). Using more bulky precursors resulted in the formation of dinuclear clusters such as [(Tp)Fe(CN)_3_Mn(TTF-salphen)·CH_3_OH],^25^ [(Tp)Fe(CN)_3_Mn(1-napen)(H_2_O)]·MeCN_3_·4H_2_O,^27^ [(Tp)Fe(CN)_3_Mn(5-Clsalen)(H_2_O)],^27^ [(Tp)Fe(CN)_3_Mn(2-acnapen)(MeOH)]·MeOH,^27^ and [(Tp)Fe(CN)_3_Mn(3-MeOsalen)(H_2_O)]^27^ in MeCN/MeOH/H_2_O solvent mixtures. On the other hand, several examples of 1D chains have been reported, including [Mn (diphenylsalen)Fe(Tp)(CN)_3_]*_n_*, formed in MeOH/MeCN, [Mn (diphenylsalen)Fe(Tp*)(CN)_3_·2H_2_O]*_n_* from MeCN,^26^ {[(Tp*)Fe(CN)_3_Mn(salcyen)]·H_2_O·2CH_3_OH}*_n_* from methanol,^18^ and [(Tp)Fe(CN)_3_Mn(2-acnapen)]·H_2_O in MeCN/H_2_O,^27^ and Hong and co-workers attributed the tendency of 1D chain formation to solvent effects on intermolecular interactions, such as face-to-face π–π contacts, and edge-to-face CH-π forces and hydrogen bonds that led to stabilizing the dimeric structure in the presence of MeOH, which blocks the evolution toward higher dimensionality 1D structures due to its coordinative nature. In the absence of MeOH, the reaction proceeds toward higher dimensionality products with less intermolecular interactions.^27^ The formation of (**1**) and (**2**) in the presence of MeOH refutes the effect of solvent in determining the product structure based on the argument of chain formation dependence on the absence of MeOH. This result indicates that this reaction system is quite sensitive to different factors besides the solvent effects on intermolecular interactions, including solvent coordinative nature and polarity, steric and electronic properties of the precursors, and reaction conditions (temperature, concentration, molar ratio, etc.). This conclusion is further supported by previously reported 1D chains isolated in the presence of MeOH such as [Mn (diphenylsalen)Fe(Tp)(CN)_3_]*_n_*, formed in MeOH/MeCN, and {[(Tp*)Fe(CN)_3_Mn(salcyen)]·H_2_O·2CH_3_OH}*_n_* from methanol.^18^

### 3.2 Crystal Description

#### 3.2.1 Crystal Description for Compound **(1)**

**Figure 1:**
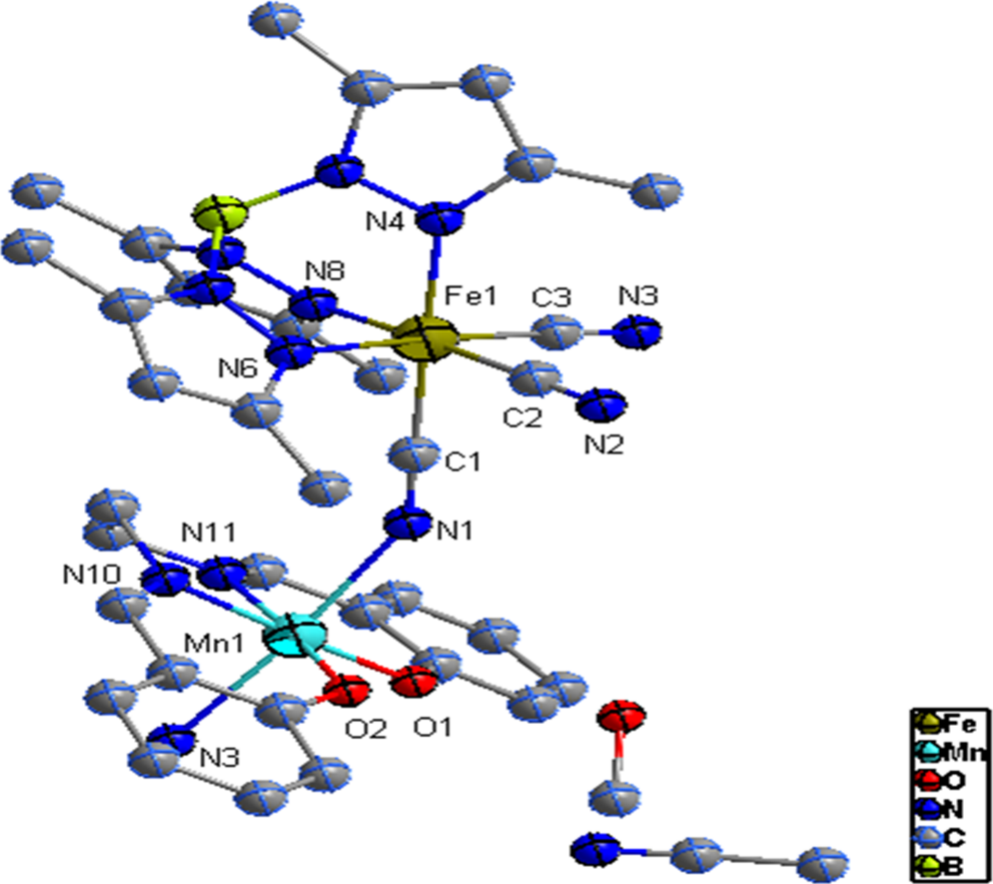
View of the asymmetric unit of **1**. Thermal ellipsoids are drawn at the 50% probability level. Hydrogen atoms are omitted for clarity.

**Figure 2:**
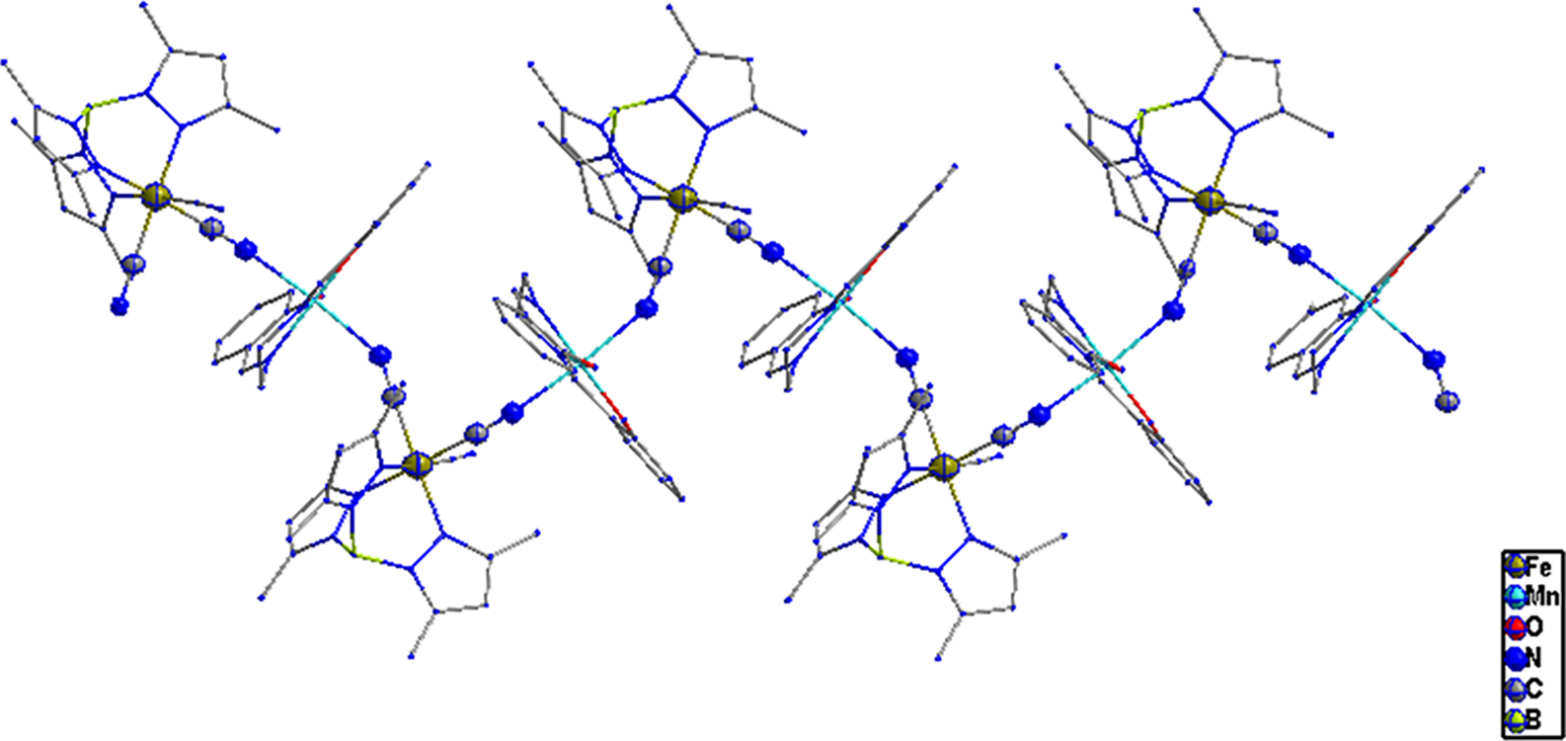
View along the *y*-axis of cyano-bridged zigzag chains in **1**.

**Figure 3:**
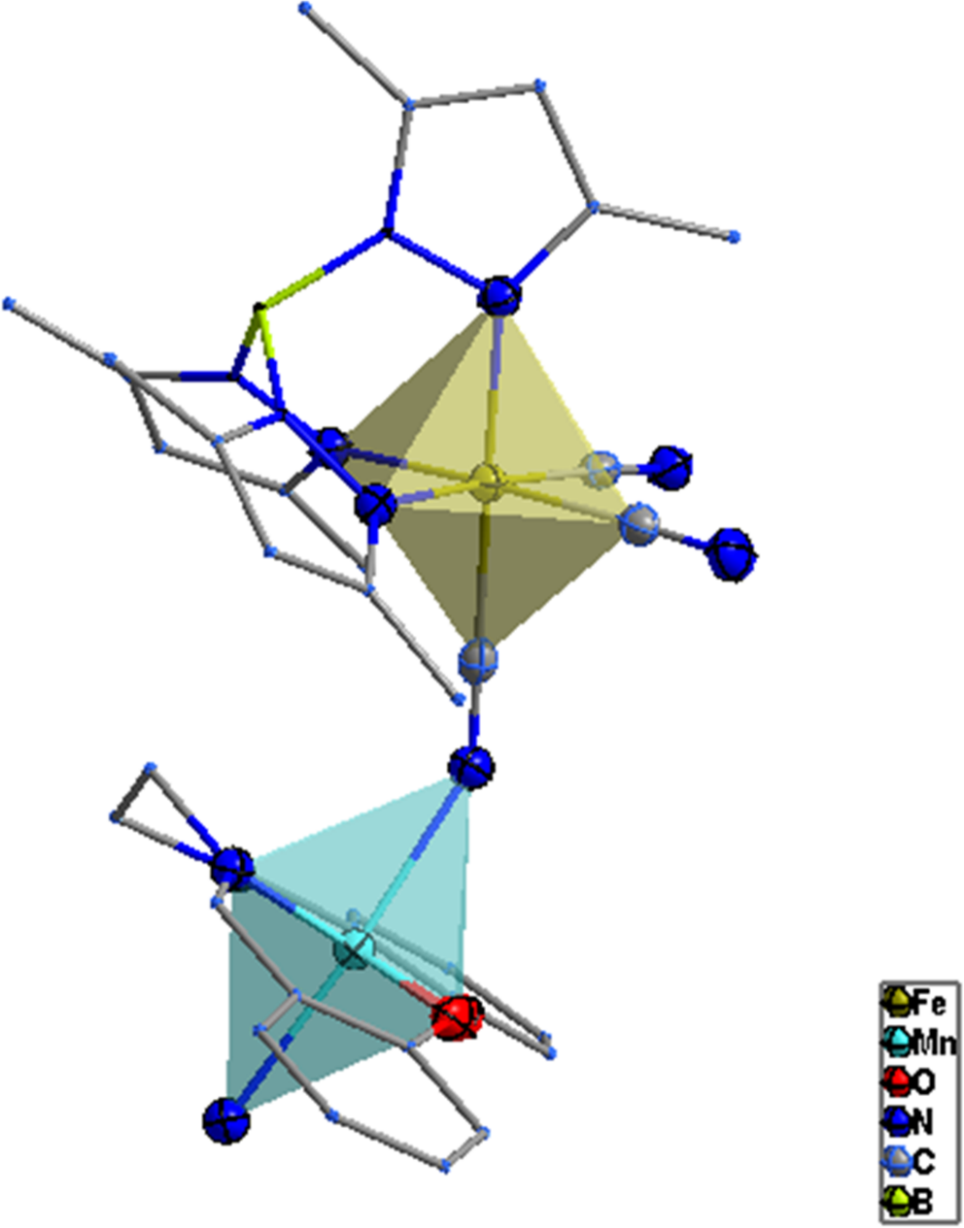
Distorted octahedral coordination geometries around Fe and Mn centers in 1.

**Figure 4:**
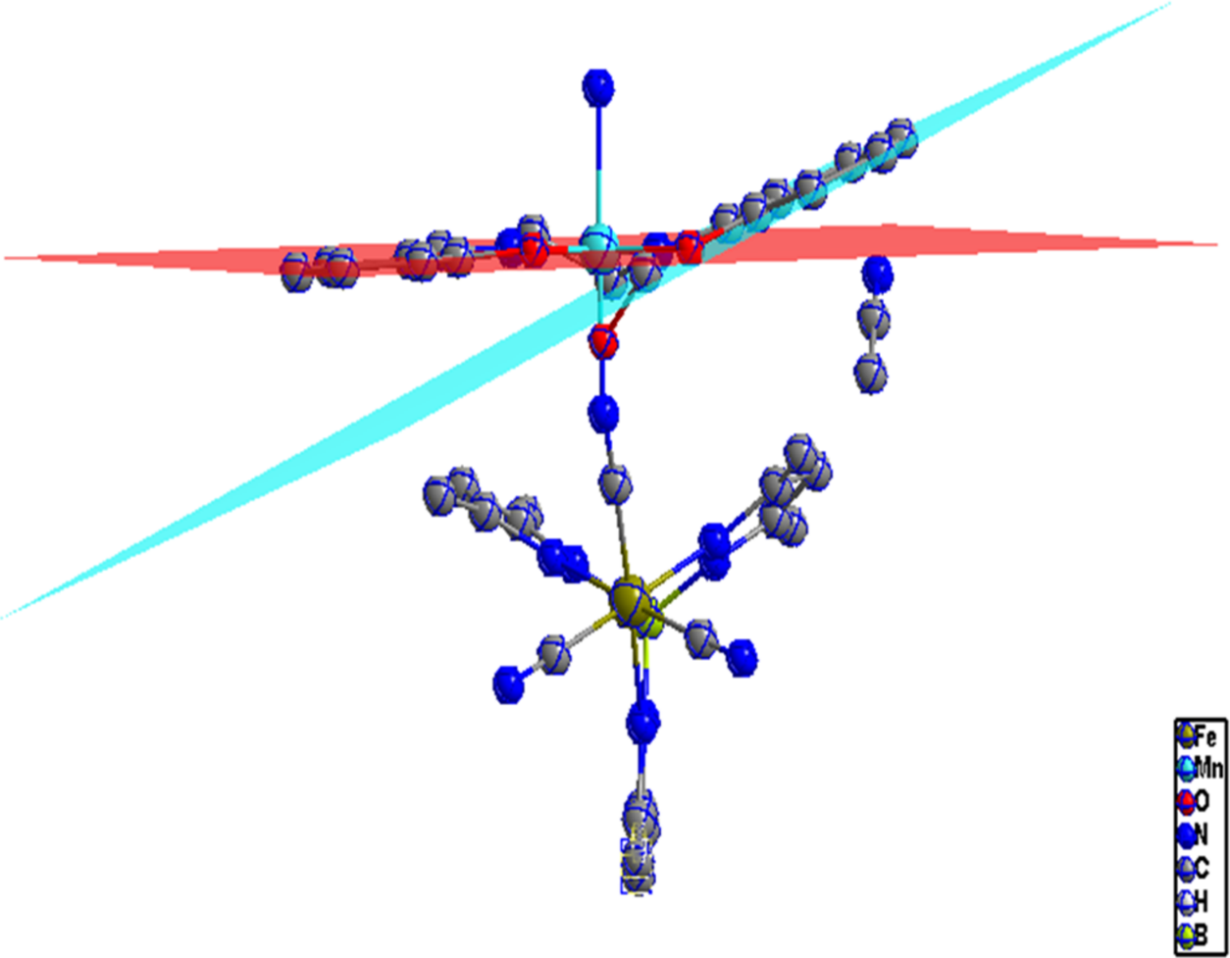
Dihedral angle between Mn and salen in **1**.

**Figure 5:**
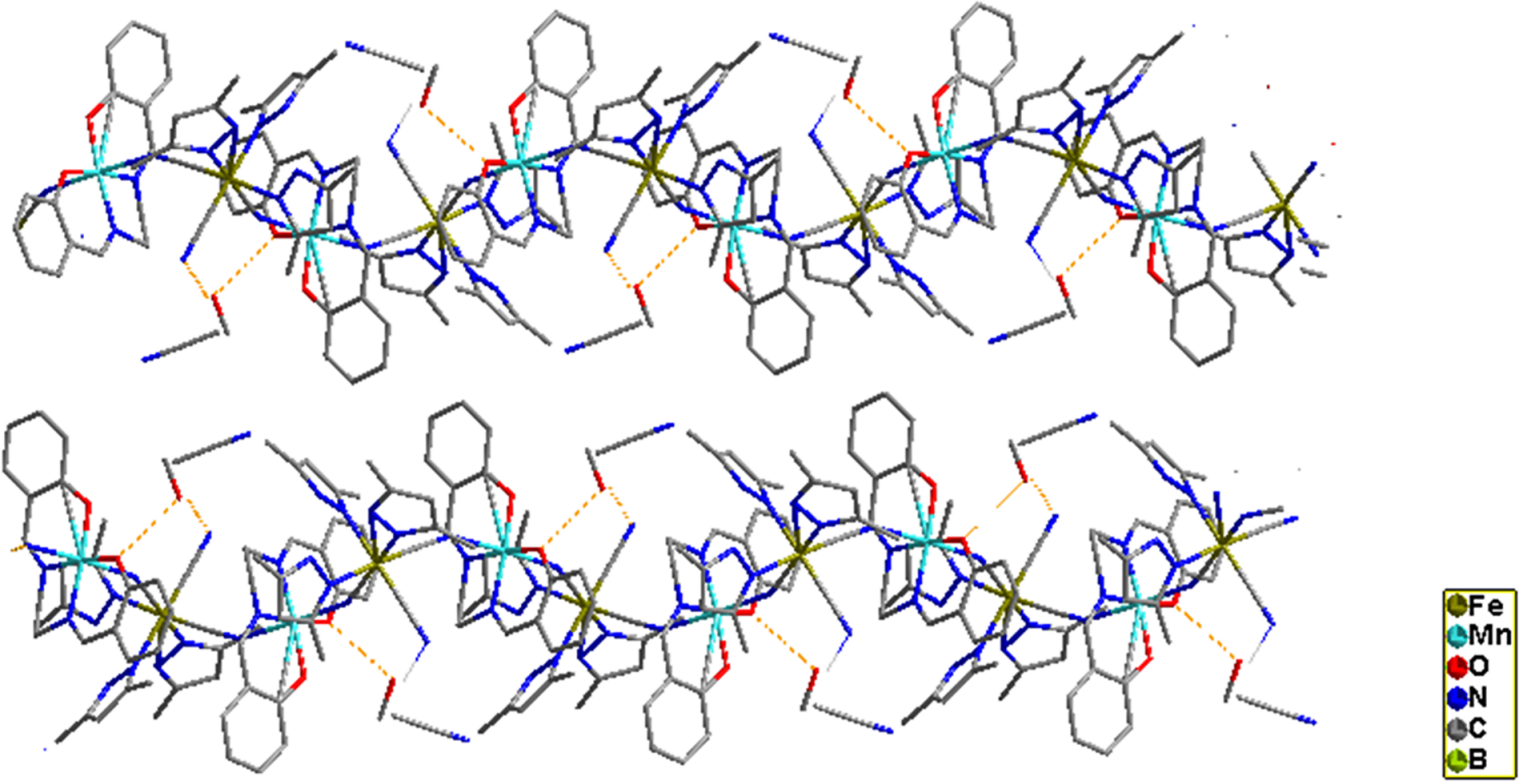
Molecular view of **1** along the *z-*axis showing H bonding between the chains and solvent molecules, which leads to further structural stability.

Structural analysis of complex **(1)** (Figure 1) showed that it crystallizes in a monoclinic space group P2_1_/n with two central metal ions (Fe, Mn) bridged via cyanide ligands within (−Fe–C≡N–Mn–N≡C−)*_n_* as the repeating unit to form 1D cyano-bridged zigzag chains (Figure 2). The structural parameters and selected bond lengths and angles are listed in Tables 1 and 2, respectively. Both metal centers are in distorted octahedral coordination environments, as shown in Figure 3. The iron center within the [Tp*Fe(CN)_3_]^−^ moiety undergoes a slight distortion with bridged cyanide bond distances (Fe–C1 = 1.908(2) Å. and Fe–C3 = 1.900(2)Å) slightly shorter than the terminal cyanide (Fe1–C2 = 1.923(2)Å). Such behavior is expected due to the bridging mode, as reported previously, where Fe^III^–C(cyano) bond lengths are in the range of 1.900(2)–1.928(2)Å.^36^ A trigonal compression along the *C*_*3*_ axis is shown within the iron tricyanide moiety with an average C–Fe–C angle equal to 85.78° as compared to an average 89.9° bite angle of the Tp* ligand. The manganese center coordination sphere consists of N_2_O_2_ donor atoms from salen ligand occupying the equatorial positions (Mn1–N10 = 1.9919(17)Å, Mn1–N11 = 1.9973(18)Å, Mn1–O1 = 1.9010(15)Å, Mn1–O2 = 1.8765(15)Å). The axial positions are occupied with nitrogen atoms from bridging cyanide ligands with longer bond distances (Mn1–N3 = 2.2269(17)Å, Mn1–N1 = 2.2467(17)Å), leading to a tetragonally elongated coordination environment due to Jan Teller distortions. These bond distances are similar to previously reported compounds.^36,27^ Iron cyanide bonding angles (Fe1–C1–N1 = 177.41(17)°) are slightly bent, whereas (Mn1–N1–C1 = 148.519(143)° and Mn–N3–C3 = 152.990(127)°) are significantly bent. The dihedral angle in Mn–salen is 21.681(24)°, as shown in Figure 4, which is typical in similar compounds.^36^ The chains are further engaged in H bonding with solvent molecules, leading to extra structural stability, Figure 5.

**Table 1:** Crystal Structure Data and Refinement Parameters for Compounds **1** and **2**

compound	**1**	**2**
empirical formula	C_37_H_42_BFeMnN_12_O_3_	C_38.67_ H_38_BFeMn N_11_ O_4_
formula weight	824.4 g/mol	842.48 g/mol
temperature/*K*	110.2	110.2
crystal system	monoclinic	orthorhombic
space group	*P*2_1_/*n*	*Pbca*
*a*/Å	12.491(3)	14.314(3)
*b*/Å	13.792(3)	20.819(4)
*c*/Å	23.351(5)	25.357(5)
*β*/°	96.11(3)	90.0
volume/Å^3^	4000.1(14) Å^3^	7556.47(300) Å^3^
*Z*	4	8
ρ_calc_g/cm^3^	1.369	1.481
μ/mm^–1^	0.73	0.776
*F*(000)	1712	3480
radiation	Mo Kα (λ = 0.71073)	Mo Kα (λ = 0.71073)
reflections collected	44688	85505
final *R* indexes [*I* > = 2σ (*I*)]	*R*_1_ = 0.0352, *wR*_2_ = 0.1010	*R*_1_ = 0.0423, *wR*_2_ = 0.1024
final *R* indexes [all data]	*R*_1_ = 0.0507, *wR*_2_ = 0.1181	*R*_1_ = 0.0616, *wR*_2_ = 0.1130
largest diff. peak/hole/e Å^–3^	0.58/–0.67	1.28/–1.01

**Table 2:** Selected Bond Distances (Å) and Bond Angles (deg) for Compounds **1** and **2**

[Tp*Fe (CN)_3_Mn^III^salen]·MeOH·MeCN (**1**)			
Fe1–N6	2.0019(17)	N6–Fe1–N8	90.38(7)
Fe1–N4	1.9748(17)	N4–Fe1–N6	90.10(7)
Fe1–N8	2.0141(1)	N4–Fe1–N8	89.09(7)
Fe1–C1	1.908(2)	C1–Fe1–N6	92.22(7)
Fe1–C2	1.923(2)	C1–Fe1–N4	177.58(7)
Fe1–C3	1.900(2)	C1–Fe1–N8	90.21(7)
Mn1–O1	1.9010(15)	C1–Fe1–C2	86.54(8)
Mn1–O2	1.8765(15)	C1–Fe1–C3	85.43(8)
Mn1–N11	1.9973(18)	C3–Fe1–C2	85.50(9)
Mn1–N10	1.9919(17)	Fe1–C1–N1	177.41(17)
Mn1–N1	2.2467(17)	Mn1–C1–N1	148.52(15)
Mn1–N3	2.2269(17)	O2–Mn1–O1	94.92(7)
C1– N1	1.1516(31)	O2–Mn1–N10	91.67(7)
C2– N2	1.1532(29)	O2–Mn1–N1	89.80(6)
C3– N3	1.1582(23)	O2–Mn1–N3	91.44(6)
		O2–Mn1–N11	172.99(7)

[Tp*Fe (CN)_3_Mn^III^salphen]·MeOH·H_**2**_O (**2**)			
Fe1–N6	2.002(2)	N6–Fe1–N8	88.24(8)
Fe1–N4	2.0159(21)	N4–Fe1–N6	90.87(8)
Fe1–N8	1.9853(21)	N4–Fe1–N8	89.13(8)
Fe1–C1	1.9038(25)	C1–Fe1–N6	92.64(9)
Fe1–C2	1.9388(26)	C1–Fe1–N4	176.27(9)
Fe1–C3	1.9413(26)	C1–Fe1–N8	92.25(9)
Mn1–O1	1.8877(18)	C1–Fe1–C2	84.76(10)
Mn1–O2	1.8821(18)	C1–Fe1–C3	88.84(10)
Mn1–N11	1.9997(21)	C3–Fe1–C2	90.313(103)
Mn1–N10	1.9927(21)	Fe1–C1–N1	178.3(2)
Mn1–N1	2.2704(22)	Mn1–C1–N1	154.9(2)
Mn1–N3	2.3057(22)	O2–Mn2–O1	91.19(8)
C1– N1	1.1537(32)	O2–Mn2–N10	174.78(8)
C2– N2	1.1547(34)	O2–Mn2–N1	93.41(8)
C3– N3	1.1542(33)	O2–Mn2–N3	92.33(8)
		O2–Mn2–N11	93.61(8)

#### 3.2.2 Crystal Structure Description for (**2**)

**Figure 6:**
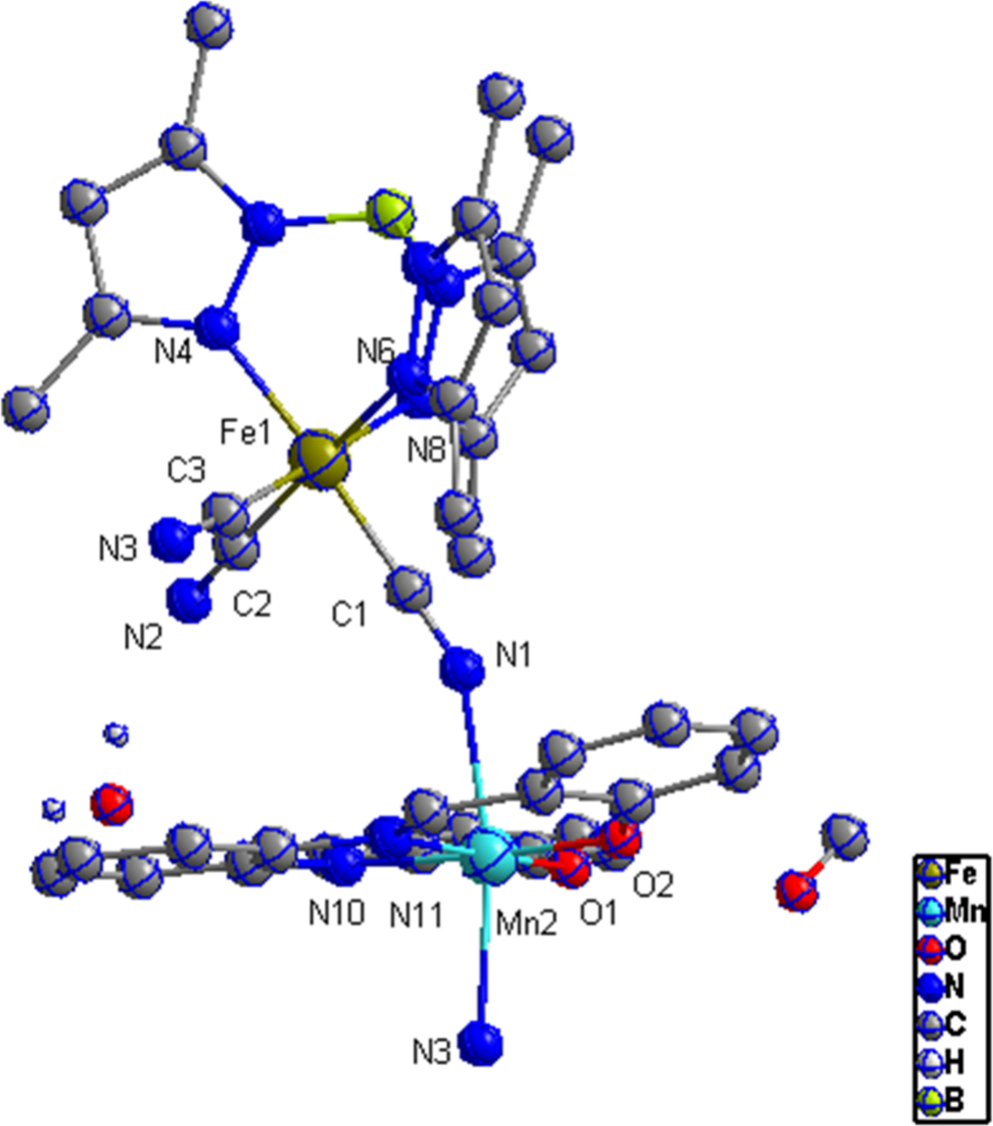
View of the asymmetric unit of complexes **2**. Thermal ellipsoids are drawn at the 50% probability level. Hydrogen atoms are omitted for clarity.

**Figure 7:**
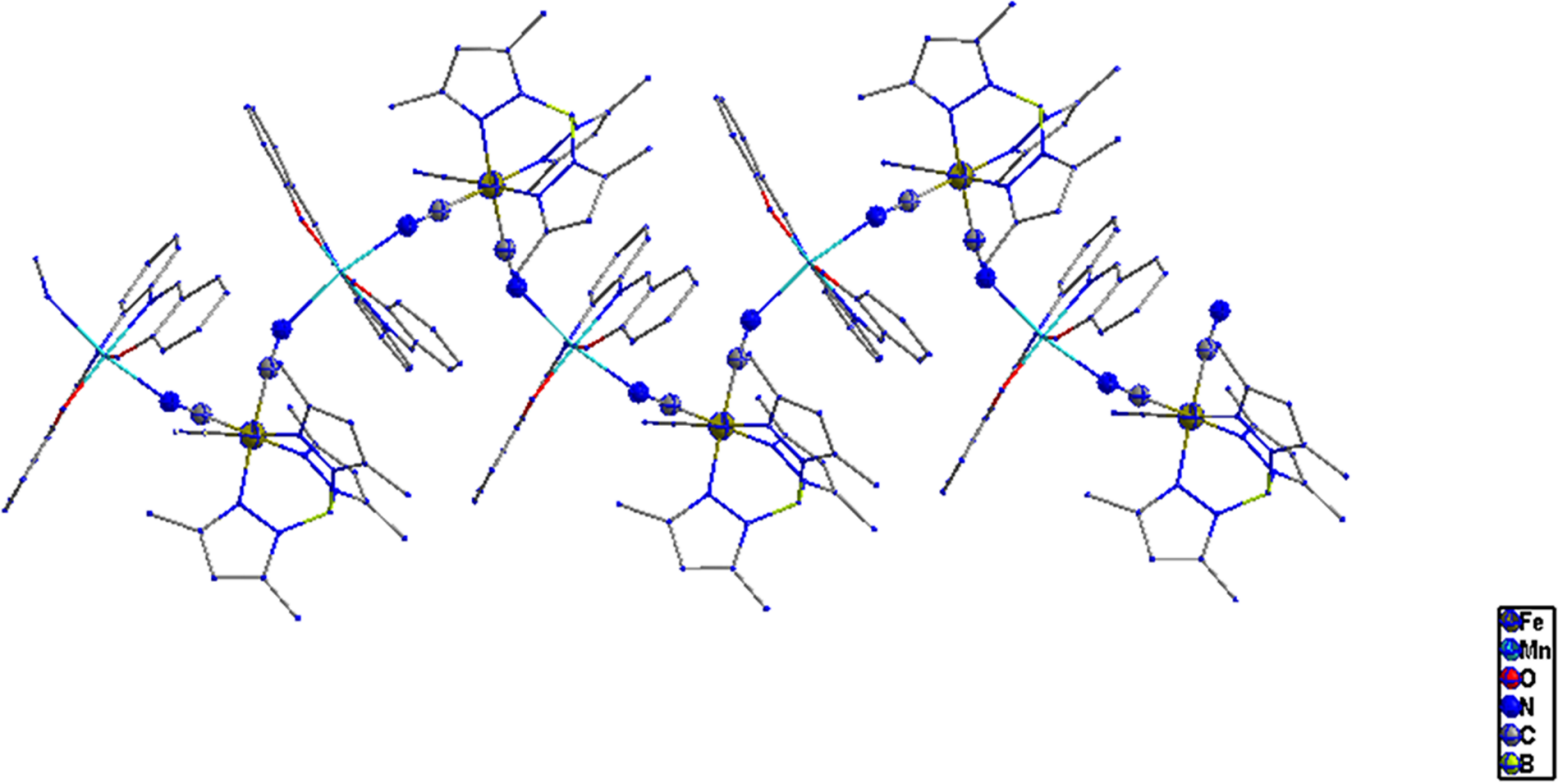
View along the *y*-axis of cyano-bridged zigzag chains in **2**.

**Figure 8:**
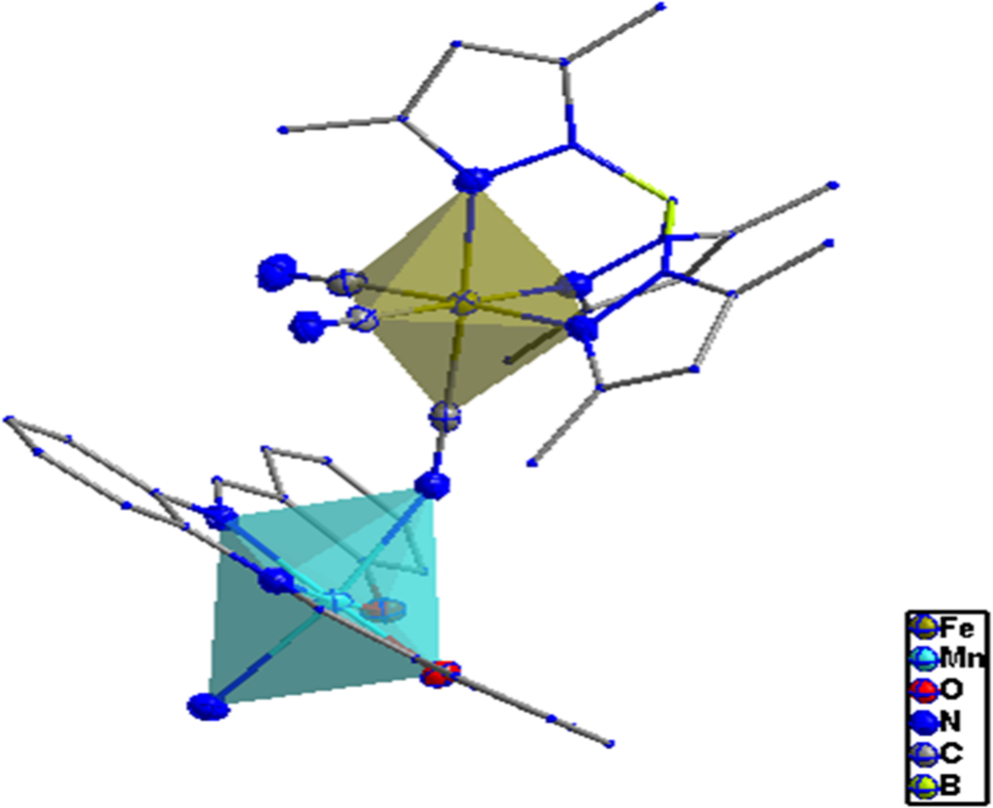
Distorted octahedral coordination geometries around the Fe and Mn centers in **2**.

**Figure 9:**
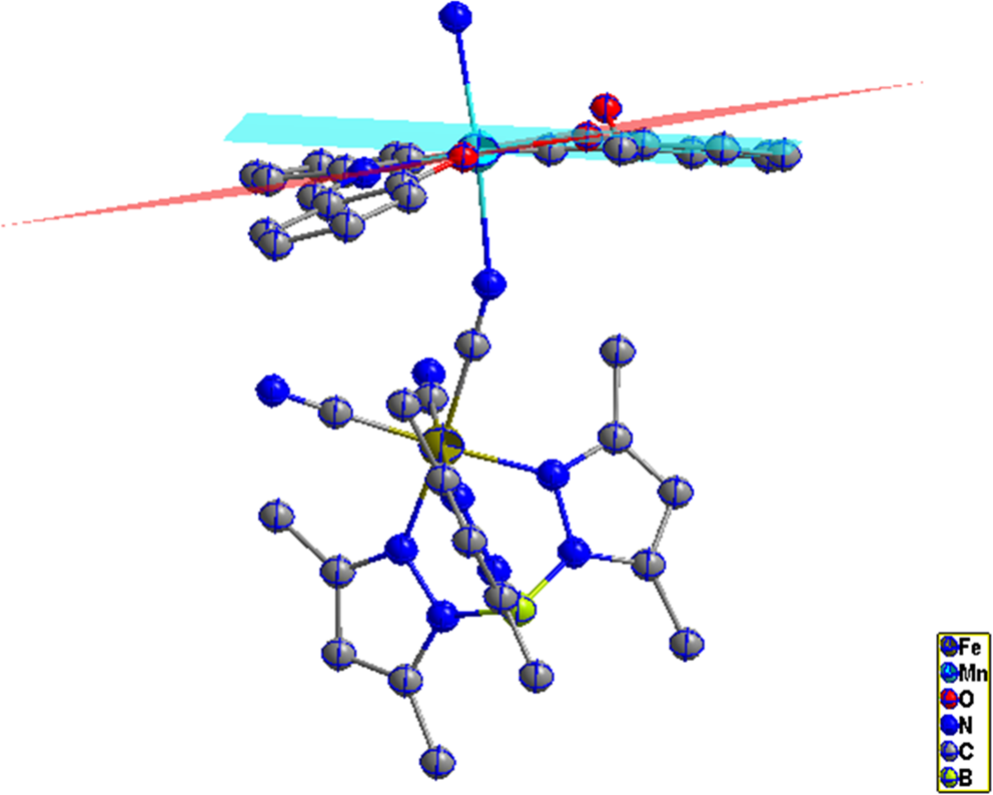
Dihedral angle between Mn and Salphen in **2**.

**Figure 10:**
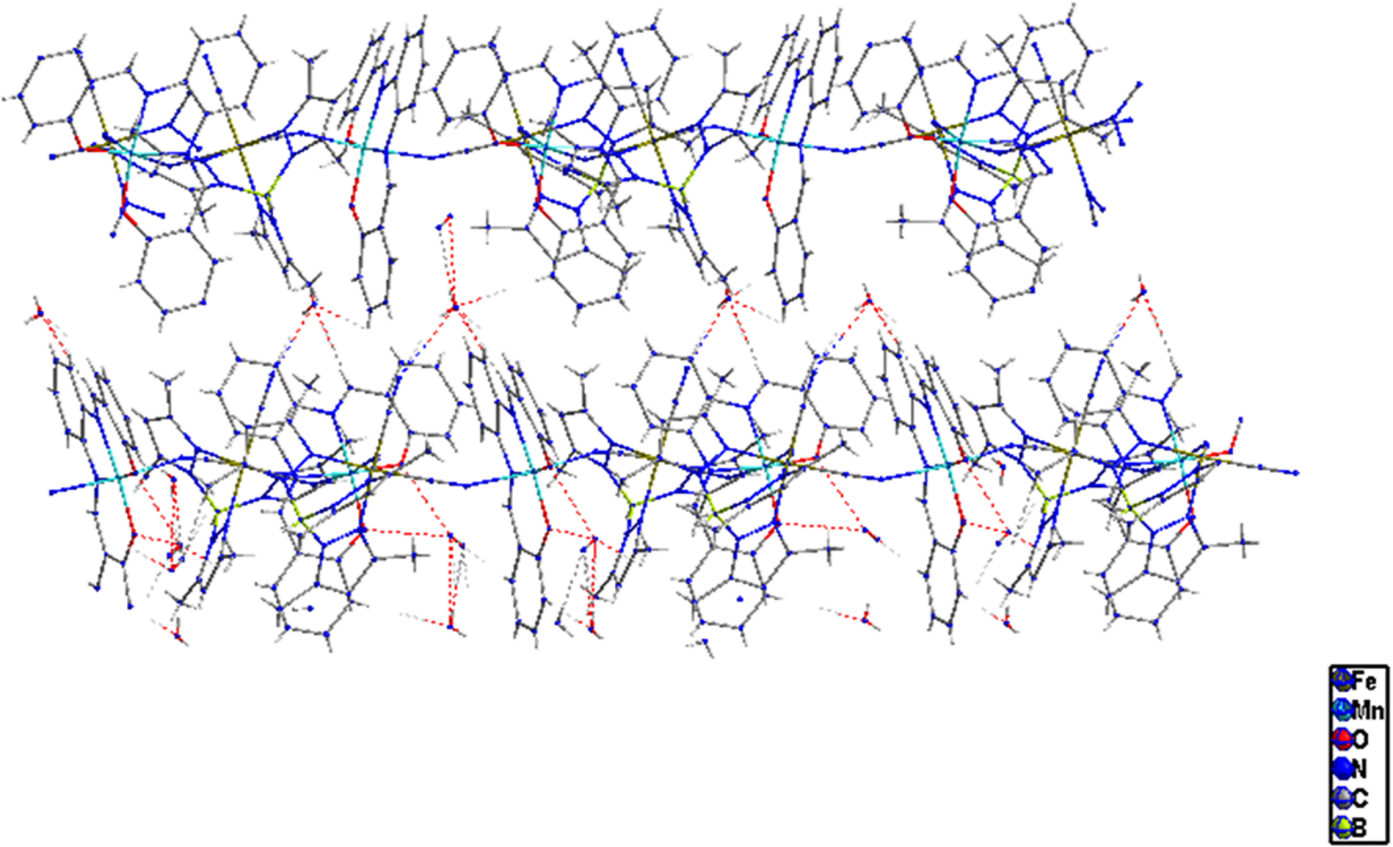
Molecular view of **2** along the *z*-axis showing H bonding between the chains and solvent molecules, which leads to further structural stability.

Structural analysis of complex **(2)** (Figure 6) showed that it crystallizes in an orthorhombic space group *Pbca* with two central metal ions (Fe, Mn) bridged via cyanide ligands within (−Fe–C≡N–Mn–N≡C−)*_n_* as the repeating unit to form 1D cyano-bridged zigzag chains, Figure 7. The structural parameters of complex **(2)** are listed in Table 1. Both metal centers are in distorted octahedral coordination environments, as shown in Figure 8. The iron center within the [Tp*Fe(CN)_3_]^−^ moiety undergoes a slight distortion with bridged cyanide bond distances (Fe–C1 = 1.9038(25))Å. and (Fe–C3 = 1.9413(26) Å) slightly shorter than the terminal cyanide (Fe1–C2 = 1.9388(26)Å). Such behavior is expected due to the bridging mode, as reported previously, where Fe^III^–C(cyano) bond lengths are in the range of 1.900(2)–1.928(2)Å.^36^ A trigonal compression along the *C*_3_ axis is shown within the iron tricyanide moiety with an average C–Fe–C angle equal to 87.971° as compared to an average 89.9° bite angle of the Tp* ligand. The manganese center coordination sphere consists of N_2_O_2_ donor atoms from the salen ligand occupying the equatorial positions (Mn1–N10 = 1.9927(21) Å, Mn1–N11 = 1.9997(21)Å, Mn1–O1 = 1.8877(18) Å, Mn1–O2 = 1.8821(18) Å). The axial positions are occupied with nitrogen atoms from bridging cyanide ligands with longer bond distances (Mn1–N3 = 2.3057(22)Å, Mn1–N1 = 2.2704(22) Å), leading to a tetragonally elongated coordination environment. These bond distances are similar to previously reported compounds. Iron cyanide bonding angles (Fe_1_–C_1_–N_1_ = 177.41(17)°) are slightly bent, whereas (Mn1–N1–C1 = 154.9(2)° and Mn1–N3–C_3_ = 151.30(19)°) are significantly bent. The dihedral angle in Mn–salphen is 12.336(208)°, as shown in Figure 9, which is typical in similar compounds.^27^ The chains are further engaged in H bonding with solvent molecules, leading to extra structural stability, Figure 10.

## 4 Magnetic Studies

**Figure 11:**
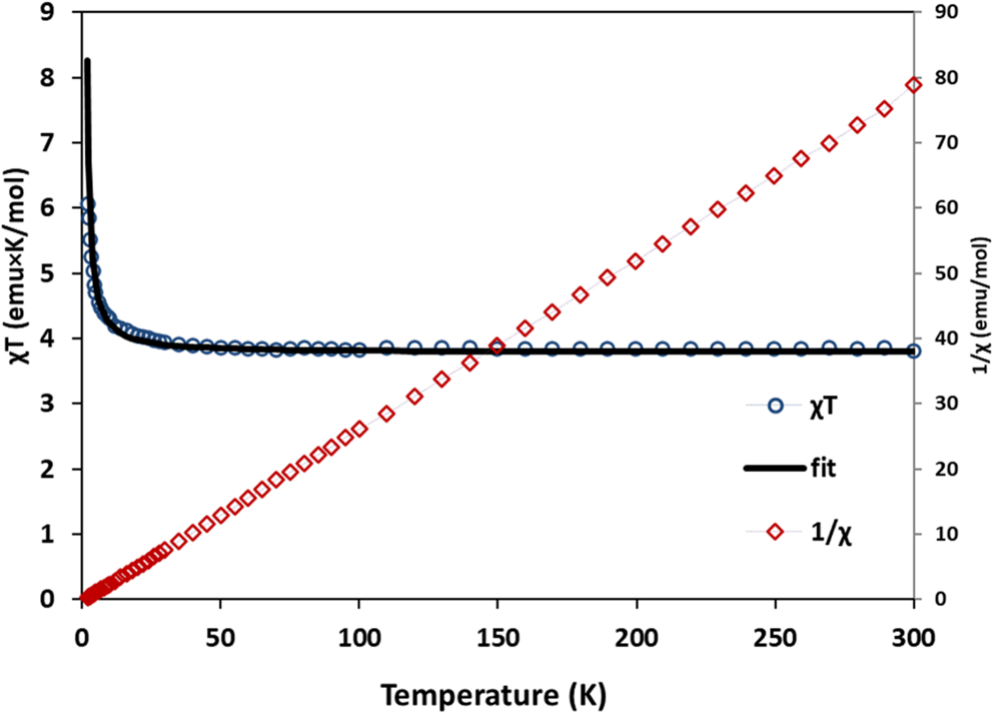
Temperature dependence of χ and χ_M_*T* of **1** in the range of 2–300 K. The solid line corresponds to the Curie–Weiss approximation with *C* = 3.76 emu·K·mol^–1^ and θ = +1.1 K.

**Figure 12:**
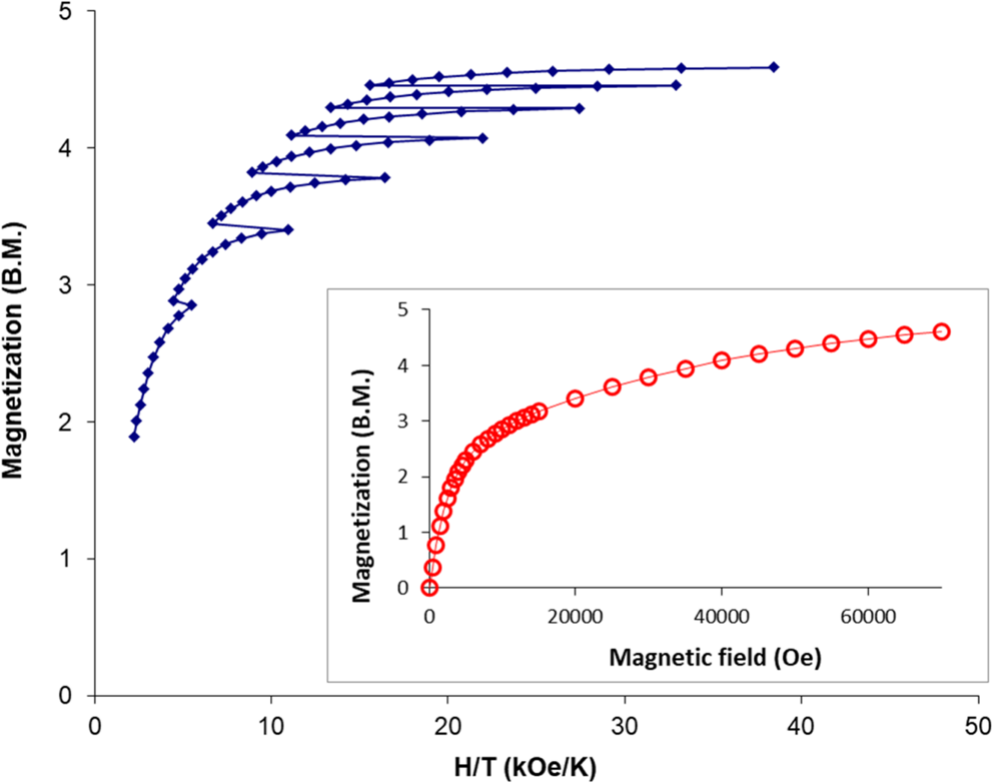
Reduced magnetization from 1.8 to 4.5 K, inset: Field dependence of the magnetization data for **1** was observed at 2 K.

**Figure 13:**
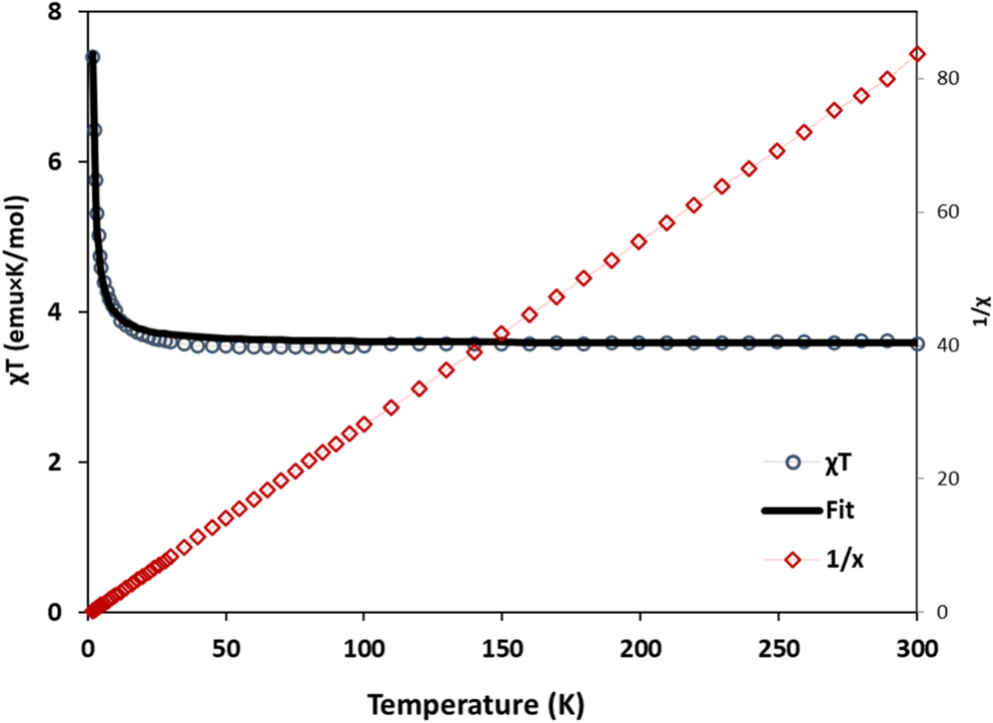
Temperature dependence of χ and χ_M_*T* of **2** in the range of 2–300 K. Solid line corresponds to curie Weiss approximation with *C* = 3.57 emu·K·mol^–1^ and θ = +1.0 K.

**Figure 14:**
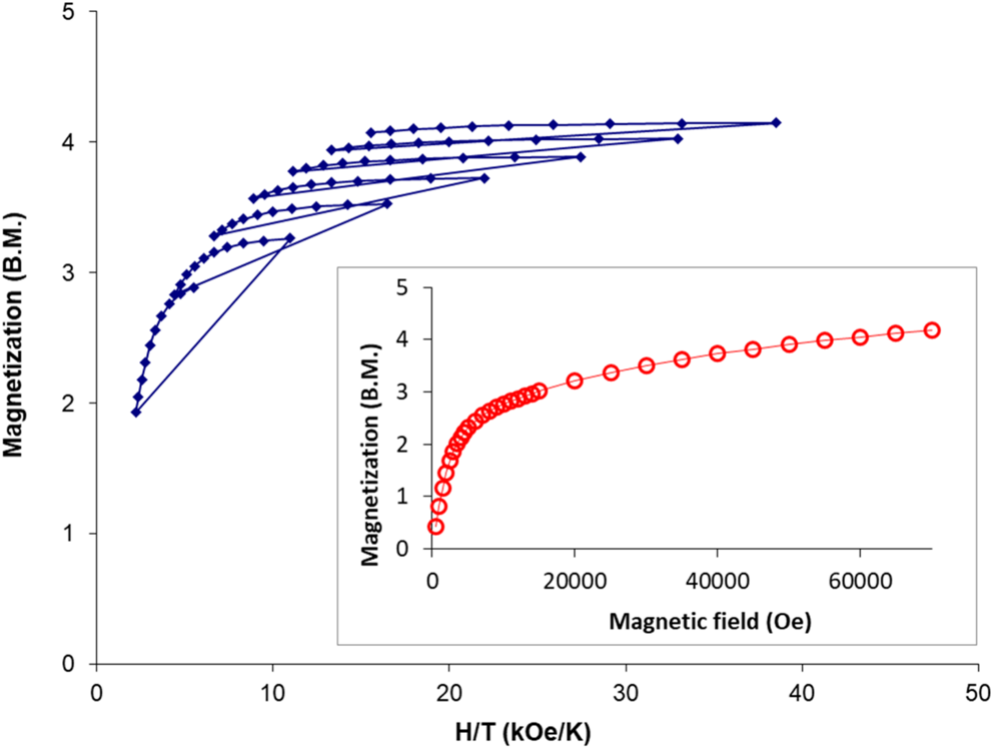
Reduced magnetization from 1.8 to 4.5 K. Inset: field dependence of the magnetization data for **2** at 2 K.

Polycrystalline samples of (**1**) and (**2**) were used to collect magnetic susceptibility measurements under a 1000 Oe dc field over the temperature range of 1.8–300 K. Room temperature χ_M_*T* values (3.8 emu·K·mol^–1^ for (**1**) and 3.61 emu·K·mol^–1^ for (**2**)) are slightly higher than the spin-only values for an *S* = 2, *g* = 2.01 Mn(III) center and *S* = 1/2, *g* = 2.8 Fe(III) (3.76 emu·K·mol^–1^ in (**1**)) and an *S* = 2, *g* = 1.95 Mn(III) center and *S* = 1/2, *g* = 2.8 Fe(III) (3.58 emu·K·mol^–1^ in (**2**)) as expected due to the anisotropy of the Fe(III) center and possible ferromagnetic coupling in both complexes (Figures 11 and 13). Upon lowering the temperature, the χ_M_*T* values exhibit a gradual increase to 6.0 emu·K·mol^–1^ (**1**) and 7.4 emu·K·mol^–1^ (**2**) at 2 K. The temperature dependence of 1/χ between 300 and 5 K in (**1**) approximates Curie–Weiss behavior with *C* = 3.76 emu·mol^–1^ K and θ = +1.1 K. Similarly, 1/χ between 300 and 5 K in (**2**) approximate Curie–Weiss behavior with *C* = 3.57 emu.mol^–1^ K and θ = +1.0 K. The positive sign of the Curie–Weiss constant suggests ferromagnetic interactions between Fe(III) and Mn(III) ions. The temperature dependence of the χ*T* product over the 2–300 K range was fit with the program PHI^33^ using the regular chain system^27^ resulting in a coupling constant *J* = +0.7 cm^–1^ (*g*_Mn_ = 2.01, *g*_Fe_ = 2.79, a temperature-independent paramagnetism (TIP) contribution of 3.0 × 10^–4^ emu·mol^–1^) for (**1**) and *J* = +0.47 cm^–1^ (*g*_Mn_ = 1.94, *g*_Fe_ = 2.67, TIP = 4.0 × 10^–4^ emu·mol^–1^) for (**2**).^37^ Ferromagnetic interactions in similar systems have been reported previously and attributed to the bent angle (Mn1–N1–C1 = 148.519(143)°, Mn1–N3–C3 = 152.990(127)° in (**1**)) and (Mn1–N1–C1 = 154.9(2)°, Mn1–N3–C3 = 151.30(19)° in (**2**)).^18,38−43^ However, linear Mn-NC angles have been reported to lead to antiferromagnetic interactions with a conversion between antiferromagnetic and ferromagnetic interactions occurring around 155°.^18^ The small magnitude of J is likely due to the reduced orbital overlap between the metal centers and the bridging ligand as further angle bending occurs. *M* versus *H* plots of the field-dependent magnetization data at fields up to 7 T were observed to saturate at values slightly lower than those expected for an isotropic *S* = 5/2 ground state for both compounds, indicating the existence of magnetic anisotropy in this system. To probe magnetic anisotropy further, the field dependence of the magnetization data at temperatures in the range of 2 and 4.5 K for **1** and **2** were collected. The iso-field lines exhibit obvious nonsuperposition, as expected for an anisotropic Fe(III) system (Figures 12, 13, and 14).

## 5 Conclusions

The combined structural and magnetic studies of (Tp*Fe (CN)_3_Mn^III^salen)·MeOH·MeCN **(1)** and (Tp*Fe (CN)_3_Mn^III^salphen)·MeOH·H_2_O **(2)** [Tp* = hydrotris(3,5-dimethylpyrazol-1-yl)borate, salen = *N*,*N*′-ethylenebis(salicylideneiminate), salphen = *N,N*-bis(salicylidene)-1,2- phenylenediamine] indicate 1D zigzag chain structure bridged via cyanide ligands within the (−Fe–C≡N–Mn–N≡C−)*_n_* motif with ferromagnetic coupling between the Fe^III^ and Mn^III^ metal centers with Curie–Weiss constants of +1.1 K in (**1**) and +1.0 K in (**2**). The formation of 1D chains in the presence of MeOH refutes previous reports that attributed chain formation to the absence of MeOH in the reaction media. Such a result suggests a highly sensitive nature of this reaction system to different factors, including solvent effects on intermolecular interactions, solvent coordinative nature and polarity, and steric and electronic properties of the precursors as well as reaction conditions (temperature, concentration, molar ratio. etc.).
